# FRET from single to multiplexed signaling events

**DOI:** 10.1007/s12551-017-0252-z

**Published:** 2017-03-23

**Authors:** Gertrude Bunt, Fred S. Wouters

**Affiliations:** 1 Clinical Optical Microscopy, Department of Neuropathology, University Medical Center Göttingen, Robert-Koch-Strasse 40, 37075 Göttingen, Germany; 2 Laboratory for Molecular and Cellular Systems, Department of Neuropathology, University Medical Center Göttingen, Waldweg 33, 37073 Göttingen, Germany; 3 DFG Research Center “Nanoscale Microscopy and Molecular Physiology of the Brain”, Göttingen, Germany

**Keywords:** FRET, Multiplexing, Transfer rate, FRET efficiency, Protein interaction, Protein complex

## Abstract

Förster resonance energy transfer (FRET) is a powerful tool for the visualization of molecular signaling events such as protein activities and interactions in cells. In its different implementations, FRET microscopy has been mainly used for monitoring single events. Recently, there has been a trend of extending FRET imaging towards the simultaneous detection of multiple events and interactions. The concomitant increase in experimental complexity requires a deeper understanding of the biophysical background of FRET. The presence of multiple acceptors for one donor affects the well-known formalism for FRET between two molecules, increasing distance sensitivity through mechanisms that have become known as the ‘antenna’ and ‘surplus’ effect. We will discuss the nature of these effects and present the imaging methods that have been used to unravel the combined transfer rates in the multi-protein interactions of multiplexed FRET experiments. Multiplexing strategies are becoming invaluable analytical tools for the elucidation of biological complexes and for the visualization of decision points in cellular signaling networks in physiological and pathological conditions.

## Introduction

When in immediate molecular proximity, the energy levels of fluorescent molecules can couple by Förster resonance energy transfer (FRET). This coupling can be measured in a microscope and provides a sensitive and robust metric for the interaction of the biomolecules carrying the fluorescent labels. The benefits for the life sciences lie in the fact that protein signaling and effector networks operate, in large parts, through conformational changes and the binding and unbinding of protein components. FRET allows the direct visualization of these decisive events through a variety of microscopy methods.

The cellular signaling network consists of multiple interconnecting and simultaneously occurring single events that, in the end, lead to a unified cellular response. FRET is used to entangle its working, but most FRET studies restrict themselves to the visualization of one signaling event by a FRET biosensor labeled with one FRET fluorophore pair. However, the correlation of several individual events over different cells is complicated by the inhomogeneity in cellular responses. For this reason, the last ten years has seen a rise in popularity in the extension of FRET measurements to the simultaneous detection of several events with multiple fluorophores and biosensors in a single cell. This ‘multiplexed’ detection has involved the parallel read-out of multiple single biosensors for interactions and signaling events, as well as structural studies on the composition of large multi-protein complexes and the detection of multiple conformations of molecules.

**Fig. 1:**
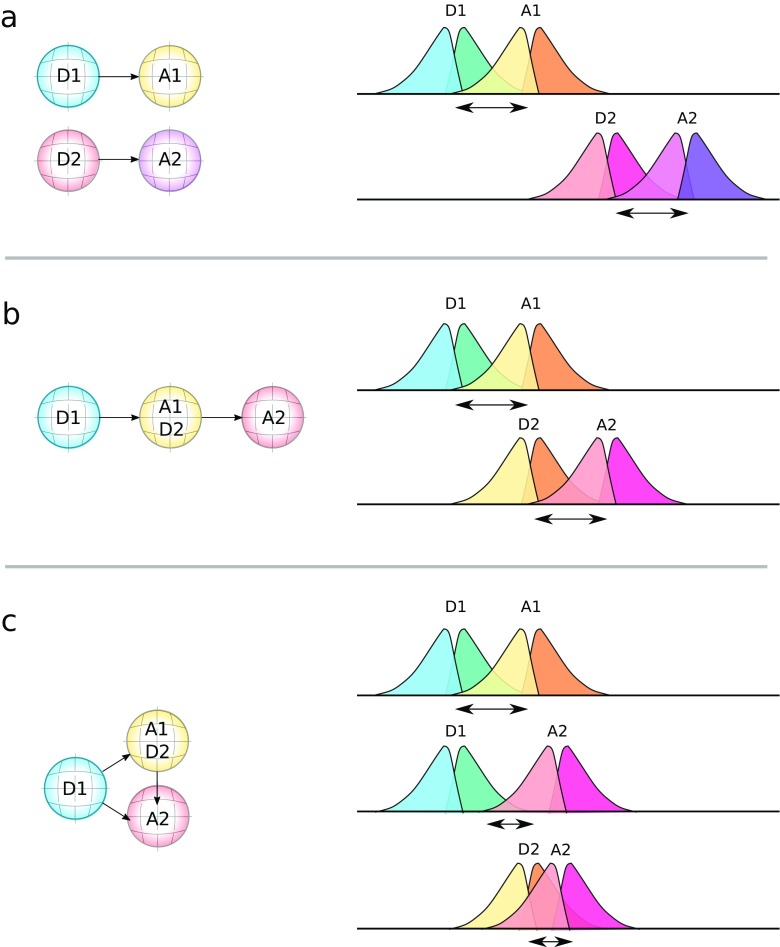
Types of Förster resonance energy transfer (FRET) multiplexing schemes. **a** Parallel FRET assays. These are typically intramolecular FRET sensors co-expressed in a cell, each containing a donor (*D*) and acceptor (*A*) fluorophore that are spectrally separable. Each sensor is read out individually in this design. **b** Sequential, two-step, FRET between three fluorophores that form two consecutive FRET donor and acceptor pairs. The acceptor of the first pair acts as the donor for the second acceptor. This design often involves conformational studies on single DNA and protein molecules that contain all three fluorophores. The D1-to-A2 FRET can span distances that are significantly greater than can be reached in a single FRET transition. **c** Three-fluorophore FRET used to detect multiple interactions in a complex, in which one donor shares coupled FRET paths with two acceptors that are usually, but not necessarily, spectrally different. FRET detection between the acceptors can be read out in addition when they form a donor–acceptor pair (D2–A2). The difference to the two-step FRET situation is that both acceptors require spectral overlap with the donor. Schematic excitation and emission spectra are shown for the donor and acceptor pairs, respectively. The *double-headed arrows* indicate the spectral overlap between donor emission and acceptor excitation spectra

Typical FRET biosensors for the detection of single events are genetically encoded intramolecular sensors consisting of a single molecule, often a protein domain labeled with one donor and one acceptor fluorophore, that responds on a conformational change or cleavage. Intermolecular FRET designs are based on two interacting proteins, each labeled with either donor or acceptor fluorophores, for the detection of protein complex formation or their modifications. Both types of sensor also form the basis for the experimental designs of FRET multiplexing (Fig. 1).

Parallelization approaches have been reported in which simultaneously ongoing signaling reactions are followed by the expression of several intramolecular FRET sensors in one cell (Fig. 1a). Such a multiplexing experiment was performed by Piljic and Schultz (2008). They managed to simultaneously monitor three calcium-dependent signaling events by the combination of a cytosolic sensor for calcium/calmodulin-dependent protein kinase IIα, a membrane-bound sensor for protein kinase C, and a translocating FRET sensor based on annexin A4. Similarly, several ratiometric intramolecular sensors for the simultaneous monitoring of changes in the intracellular levels of various second messengers (Niino et al. 2009, 2010) and protein kinase A (PKA) activity (Aye-Han et al. 2012; Woehler 2013) were detected in parallel.

Further studies show the detection of combinations of enzymes and other small cellular analytes in the same cell, e.g., Src and MT1-MMP activities (Ouyang et al. 2010); caspase-3 activity and Ca^2+^ dynamics (Ding et al. 2011); and caspase-3 activity together with pH and redox co-factors (Sergeeva et al. 2017). In all these studies, the read-out of the individual sensors is based on the spectral separation of compatible and orthogonal FRET pairs.

To overcome spectral contamination, a multi-read-out approach using the additional dimension of fluorescence lifetimes in combination with spectral separation was used to discriminate Ras activity from a spectral ratiometric chameleon Ca^2+^ sensor (Grant et al. 2008). Another multiparameter study detected fluorescent lifetime changes of TN-L15, a genetic FRET calcium sensor, alongside homo-FRET detection by anisotropy of an AKT domain as an indicator of 3′-phosphoinositide accumulation (Warren et al. 2015). Recently, both the spectral and lifetime characteristics of a newly developed FRET donor, the monomeric cyan-excitable red fluorescent protein (mCyRFP1), were exploited to simultaneously monitor two signaling events (Laviv et al. 2016).

Irrespective of the popularity of the parallel approach of intramolecular sensors, other experimental designs for the detection of multiple conformational and intermolecular protein interactions are under active development. An extension of the multiplexing designs for intramolecular reporters involves consecutive ‘three-fluorophore’ or ‘two-step’ FRET (Maliwal et al. 2012; Watrob et al. 2003) (Fig. 1b). This approach has been used to address multiple conformational states within a molecule. In this case, one single molecule contains three fluorophores that form two consecutive FRET pairs. Furthermore, switching FRET was used to probe multiple distances in protein–DNA complexes (Uphoff et al. 2010). These studies mostly involve single-molecule FRET detection (smFRET) in order to circumvent the inhomogeneity of the reaction that complicates the interpretation in ensemble measurements.

Intermolecular ensemble measurements for multiple FRET detection by ‘three-fluorophore’ FRET using separate labeled proteins have predominantly involved the visualization of up to three interactions in a protein complex. Here, up to three individual protein components are each labeled with one fluorophore, which undergo combined energy transfer between them, such as the combination cyan, yellow, and red fluorescent proteins (Fig. 1c).

The first investigation of this kind in living cells was by Galperin et al. (2004), who showed the ternary interaction of proteins in Rab5-EEA.1 microdomains of endosomes and the interaction of EGFR with the adaptor protein Grb2 and the tyrosine phosphoprotein Cbl. A similar approach was then used to investigate the dimerization of the transcription factor C/EBPα and its interaction with the heterochromatin protein 1α (HP1α) (Sun et al. 2010).

Other ‘three-way’ or ‘triple-color’ FRET investigations were used to resolve binary interactions for the following set of protein triples: the adapter proteins SLP-76, Nck, and the guaninine nucleotide exchange factor Vav1 (Pauker et al. 2012); the actin-regulating complex consisting of WIP and WASp with the kinase PKCθ (Fried et al. 2014); and a small actin-regulating network involving N-WASP, actin, IQGAP1, and the small Rac1 and Cdc42 G-proteins (Wallrabe et al. 2015). Using spectral unmixing and deconvolution, the multiple protein interactions of HIV viral-like particles were imaged in three dimensions using fusion constructs of the HIV-Gag protein (Scott and Hoppe 2016).

Another major application of multi-fluorophore FRET is the determination of the stoichiometries of protein complexes. The biologically relevant metric in these cases is the quantity of complexes of a defined composition in a cell. Using spectral unmixing, FRET coupling in mixed donor–acceptor complexes was resolved (Raicu 2007; Singh et al. 2013; Stoneman et al. 2016).

The challenges of multiplexing FRET approaches lie, on the one hand, in expanding the number of different fluorophores that can be combined and, on the other hand, on the interpretation of the FRET data. Perhaps the popularity of the parallel use of single-molecule reporters in a multiplexing design, despite the technical challenges of increased spectral contamination, lies in the relatively easy accessibility of the FRET responses and their interpretation, as each sensor can be addressed individually. A more complicated situation presents itself for the detection of several protein conformations or several interactions in protein complexes, as the individual FRET responses likely influence each other. Nevertheless, these sensing schemes can provide unique event coincidence and stoichiometric information. The increasing complexity in the FRET detection and analysis that accompanies the development of such sophisticated multiplexing designs requires a deeper insight into the biophysics of the FRET phenomena and its detection methods.

## Background: FRET between two molecules

FRET is the non-radiative transfer of excited state energy by dipole coupling between fluorophores that are in extreme close vicinity. For molecules with matching energies, resonance between the electron shells of a donating and accepting molecule causes the transfer of excited state energy. As a consequence, the donating molecule returns to the energetic ground state and the accepting molecule is raised to the excited state without being excited directly.

The nature of the coupling process is extremely dependent on distance, limiting FRET to short distances of up to maximally 10 nm for most biologically relevant fluorophores. This is the same order of magnitude as the length scale of proteins. Its extreme distance dependence and short range make FRET an ideal tool for probing protein–protein interactions, modifications, and conformational changes.

It was Förster’s seminal contribution to realize that the probability for two molecules to possess matching vibrational energy levels depends on the product of their frequency spectra, i.e., the degree of overlap between the donor emission spectrum and the acceptor absorption spectrum (Förster 1946, 1948). He introduced a transition probability function called the ‘overlap integral’, which describes the density of states of the excited donor and the acceptor that may be coupled and undergo isoenergetic energy transfer.

The overlap integral was the missing puzzle piece on the route to the FRET equation (Förster 1951) that is used today to predict and quantitatively describe FRET coupling with high precision. This equation (Eq. 1) describes the rate of energy transfer *k*_*T*_ from the excited donor to the acceptor, as can be obtained from experimentally accessible variables:

1 $$ {k}_T(r)=\frac{9 ln10{\kappa}^2{Q}_D}{128{\pi}^5{n}^4{N}_A{r}^6{\tau}_D}{\displaystyle \underset{0}{\overset{\infty }{\int }}}{f}_D\left(\lambda \right){\epsilon}_A\left(\lambda \right){\lambda}^4 d\lambda $$

in which *κ* ^2^ is a geometrical factor describing the relative orientation between the donor and acceptor transition dipoles, *Q*_*D*_ is the donor quantum yield in the absence of FRET, *n* is the refractive index of the medium, *N*_*A*_ is Avogadro’s number, *r* is the fluorophore separation distance, *τ*_*D*_ is the donor lifetime in the absence of FRET, *f*_*D*_(*λ*) is the fluorescence spectrum, normalized to unity, and *ϵ*_*A*_(*λ*) is the acceptor absorption spectrum normalized to its molar extinction coefficient.

We will not expand on the derivation or historical background of this famous equation, for which we refer to van der Meer (2013) and Wouters (2013). However, it is important to discuss some of its components and their relation to the energy transfer rate, as these have direct consequences for FRET coupling between two molecules and for the expansion of FRET theory to include multiple acceptors per donor, as is the case for many implementations of multiplexed FRET.

### Energy transfer rate, FRET efficiency, and lifetime

The energy transfer rate *k*_*T*_ (in units s^−1^) describes the probability of the donor de-excitation (‘decay’) by losing energy through FRET. The other decay paths are through emission of a photon, as given by the radiative rate *Γ*, and through non-radiative energy losses by interactions with the environment, as given by the non-radiative rate *k*_*nr*_.

The fraction of all decay transitions to occur through FRET is given by the FRET efficiency *E* (ranging from 0 to 1, or 100%). This is equal to the ratio of the transfer rate over all rates combined (Eq. 2). The inverse of the sum of all rates is the (average) time it takes the excited state to return to the ground state. This is called the lifetime *τ*.

2 $$ E=\frac{k_T}{k_T+\varGamma +{k}_{nr}}={k}_T{\tau}_{FRET} $$

The lifetime of the excited state of the donor (*τ*_*D,no FRET*_) is the inverse of the sum of the radiative, non-radiative, and—when present—energy transfer rate (*τ*_*D,FRET*_) (Eq. 3):

3 $$ \begin{array}{l}{\tau}_{D, NO\kern.2em  FRET}=\frac{1}{\varGamma +{k}_{nr}}\kern1em >\kern1em {\tau}_{D, FRET}=\frac{1}{k_T+\varGamma +{k}_{nr}}\\ {}{\tau}_{D, FRET}=\left(1- E\right)\cdot {\tau}_{D, NO\kern.2em  FRET}\kern1em \\ {}\kern1em \\ {} and\kern0.5em {k}_T=\frac{E}{\tau_{D, NO\kern.2em  FRET}\kern.2em \left(1- E\right)}\kern1em \end{array} $$

The donor lifetime scales with the FRET efficiency. When the transfer rate increases, the FRET efficiency increases, and the time the donor spends in the excited state decreases. Lifetime measurements, therefore, provide direct insight into the de-excitation rates that act on the donor.

### Interfluorophore distance and orientation

The Förster equation (Eq. 1) shows that FRET possesses a sixth-order dependence on separation distance. The typical distance range for FRET between a donor and acceptor is 1–10 nm. The distance dependence of FRET in a given fluorophore pair can be expressed in a critical also called Förster distance *R*_0_. This is the distance *r* = *R*_0_ at which the transfer rate equals the radiative plus non-radiative rate, and where the FRET efficiency is 0.5 (50%). From the Förster equation, this distance equals:

4 $$ {R}_0=\sqrt[6]{\frac{9 ln10{\kappa}^2{Q}_D}{128{\pi}^5{n}^4{N}_A}{\displaystyle \underset{0}{\overset{\infty }{\int }}}{f}_D\left(\lambda \right){\epsilon}_A\left(\lambda \right){\lambda}^4 d\lambda} $$

so that the transfer rate and FRET efficiency can be expressed in terms of distances:

5 $$ {k}_T=\frac{1}{\tau_D}\frac{R_0^6}{r^6},\kern0.5em  E=\frac{R_0^6}{r^6+{R}_0^6} $$

These equations (Eq. 5) show why FRET is often called a ‘molecular ruler’, as the FRET rate and efficiency depend very sensitively on distance on a molecular length scale.

**Fig. 2:**
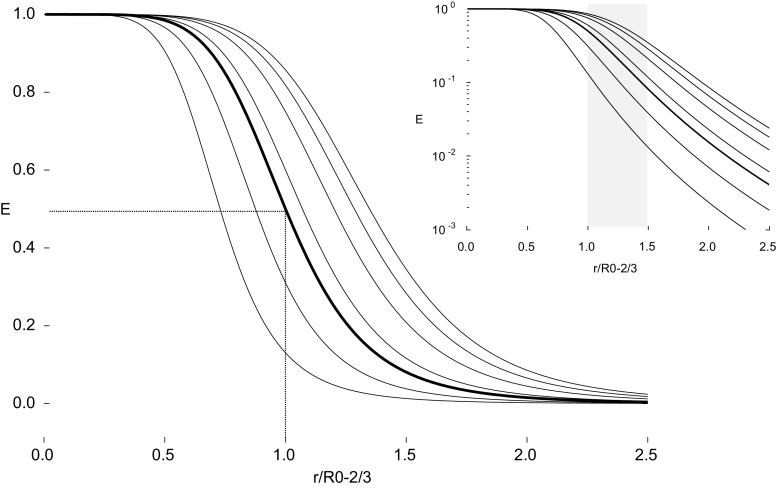
The dependence of *R*_0_ on the relative dipole orientation *κ* ^2^. FRET efficiencies (E) for fluorophore separation distances *r* are expressed relative to the Förster distance *R*_0_ assuming ${\kappa}^2=\frac{2}{3}$ (in **bold**). The *thin lines* represent the conditions for *κ* ^2^ = 0.1, 0.3, 1, 2, 3, or 4 (from left to right). The *insert* shows the same FRET efficiency curves in logarithmic scale. The *gray area* indicates the range of typical FRET efficiency values in ensemble microscopy measurements, e.g., for ${\kappa}^2=\frac{2}{3}$, a 10% change in distance results in a 40% change in FRET efficiency

Realistic experimental values for FRET efficiencies in the life sciences typically lie between ∼50 and 5%, i.e., ${R}_0\le r\le 1\frac{1}{2}{R}_0$ (Fig. 2). As *R*_0_ for common FRET pairs lies around 5 nm, a 5% change in FRET efficiency, which can be detected with confidence in FRET microscopy, corresponds to distance changes in the range of Angstroms.

A word of caution, however, is in order when distances are calculated from FRET efficiencies. The reason is that *R*_0_ depends on the relative dipole orientation *κ* ^2^ (Fig. 2). The exact value(s) of *κ* ^2^ between interacting fluorophores in an experiment is not known a priori, but its limits can be estimated from the depolarization of fluorescence (Dale et al. 1979). With this information, accurate distance measurements can be obtained. Its value depends on the angles between the donor and acceptor dipoles and ranges from 0, for a perpendicular orientation, to 4 for a collinear orientation.

A value of ${\kappa}^2=\frac{2}{3}$ is typically used for the calculation of *R*_0_ of a FRET pair, as this corresponds to the statistical average of all possible orientations. Strictly speaking, this assumption only holds when the fluorophores are entirely rotationally free. However, a value for ${\kappa}^2 = \frac{2}{3}$ is also acceptable in a regime in which the orientation of at least one of the fluorophores is fully random or mobile and leads to a small error in most cases (van der Meer 2002). At high degrees of immobility, however, distance estimations can suffer from large errors.

An instructive example of the influence of *κ* ^2^ on the FRET efficiency is the CFP-YFP fusion construct Cy11.5, in which the last 11 amino acids of CFP and the first five of YFP were removed. This head-to-tail construct has an expected interchromophore distance equal to the long axis of the fluorescent protein barrel, i.e., ∼4 nm, but sports a FRET efficiency of 98% (Shimozono et al. 2006). This high FRET efficiency cannot be explained from distances and the underlying cause should, rather, be sought in a preferred orientation of the fluorophores with *κ* ^2^ close to 4 (see also Fig. 2).

Therefore, especially for large fluorescent proteins (∼27 kDa) that are genetically fused to a protein of interest, caution should be exercised. Nevertheless, the distance dependence is on a scale that fully permits the binary detection of protein interactions and conformational changes. Considering its extreme distance sensitivity on the sub-nanometer scale, FRET microscopy can be counted as one of the optical super-resolution microscopy techniques.

## FRET with multiple acceptors: going beyond one-on-one

The Förster equation (Eq. 1) holds true for the transfer of energy from one donor to one acceptor. In FRET experiments that use multiple fluorophores, it no longer accurately describes the distance dependence and magnitude of FRET, with consequences for its quantification.

### Antenna effect: increase in distance sensitivity

**Fig. 3:**
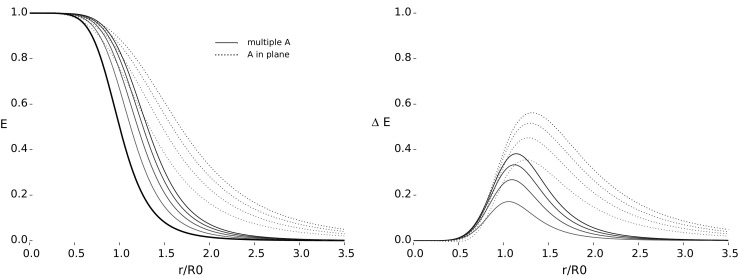
The distance sensitivity of FRET is dependent on the number of acceptors. FRET efficiencies (E) for fluorophore separation distances (*r*/*R*_0_) for a single acceptor (**bold**), for multiple acceptors at equal distance (*thin lines*, *n* = 2–5 from left to right), and acceptors at different densities *ρ* in a plane (*dotted lines*, *ρ* = 2–5 acceptors per square nanometer). The figure on the right shows the net increase for these conditions relative to the single acceptor case

**Fig. 4:**
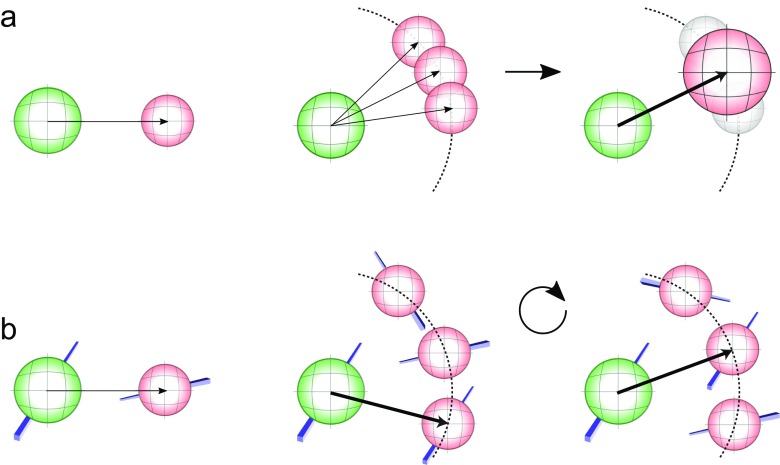
FRET ‘antenna’ and ‘surplus’ effects in the case of multiple acceptors per donor. **a** The ‘antenna’ effect. FRET increases with an increase in the number of acceptors. The multiple acceptors can be envisaged to form a new virtual acceptor that permits a higher transfer rate equal to the sum of the individual transfer rates and possesses a larger Förster distance. The sensitivity for FRET is increased at larger distances. **b** The FRET ‘surplus’ effect. For randomly distributed donors and acceptors, the average relative dipole orientation factor *κ* ^2^ for a single donor-to-acceptor FRET transition is 2/3. When the donor is presented with multiple acceptors, the probability of selecting an acceptor for energy transfer at every fluorescence transition is biased toward the acceptor molecule that exhibits a more favorable relative dipole orientation with the donor. This selection bias causes an increase of the average relative dipole orientation to exceed the 2/3 value for a single pair, causing an additional increase in the FRET rate, known as the ‘surplus’ effect. (Panel **a** was adapted and reproduced from Wouters (2013). Copyright Wiley-VCH Verlag GmbH & Co. KGaA. Reproduced with permission.)

When more than one acceptor is within reach of a donor, the probability for FRET coupling increases with the number of acceptors. For *n* identical equidistant acceptors with a single donor, *R*_0_^6^ effectively multiplies by *n*, leading to a change of (1/*n*)*r* ^6^ in the distance term. As a consequence, the distance sensitivity of FRET increases with the presence of multiple acceptors per donor without a real change in molecular distance (Fábián et al. 2010; Maliwal et al. 2012; Walczewska-Szewc et al. 2013 (Figs. 3 and 4a).

This distance-dependent increase is called the ‘antenna’ effect, as it increases the ‘sensitivity of reception’ of the donor energy. Clearly, for non-identical acceptors, the effect depends on the distances and *R*_0_ values of the individual participating acceptors. Furthermore, one should keep in mind that the antenna effect is not limited to multiplexing experiments, but can also occur in intermolecular single-sensor FRET cases in which antibodies or probes that are labeled with multiple organic dyes are used, or where oligomers are formed.

A special limiting case of the antenna effect is for a donor approaching an (infinite) planar distribution of acceptors, e.g., for biomembranes. The distance dependence now assumes a fourth power relationship with the distance of approach and depends on the density of acceptors *ρ* in the membrane. In this case, the distance term becomes (2/*πρ*)*r* ^4^, with *ρ* in the number of acceptors per square nanometer when *R*_0_ is in nanometers (Bastiaens et al. 1990; Kuhn 1972; Wouters 2013) (Fig. 3).

The increased distance sensitivity due to multiple acceptors becomes particularly apparent at distances between 1 and 1.5 times *R*_0_, i.e., the typical distance range in FRET microscopy measurements. Especially for the planar condition, the main utility of FRET, its extreme distance dependence on the molecular scale, is threatened as it adds significant sensitivity in this lower FRET efficiency range. In membranes, acceptor expression levels can be reached that severely distort the familiar highly stringent distance relationship.

The antenna effect is reflected in the resulting transfer rate that equals the sum of the individual transfer rates, the so-called ‘kinetic model’ of FRET with multiple acceptors. It also increases the FRET efficiency.

6 $$ \begin{array}{l}{k}_{T, multipleA}={k}_{T1}+{k}_{T2}\dots +{k}_{T n}\hfill \\ {}{E}_{multipleA}=\frac{k_{T1}+{k}_{T2}\dots +{k}_{T n}}{k_{T1}+{k}_{T2}\dots +{k}_{T n}+\varGamma +{k}_{nr}}\hfill \end{array} $$

At this point, it is already noteworthy to mention that the kinetic model illustrates the difference between FRET efficiencies and transfer rates (Eq. 6) as an experimental measure for energy transfer with multiple acceptors. Transfer rates from individual *D* → *A*_1_ and *D* → *A*_2_ measurements add to produce the transfer rate in the constellation *A*_1_ ← *D* → *A*_2_. In contrast, the measured, apparent, FRET efficiencies for the single pairs do not add and the sum will not be equal to the measured FRET efficiency of both acceptors. For biological applications in which the donor might couple with a single or both partners, or switch between these modes, transfer rates are, therefore, useful parameters, as they are additive and linear with acceptor coupling.

### FRET surplus

In addition to the antenna effect, another phenomenon appears to increase the FRET efficiency even further. When using constructs containing multiple Venus acceptors for a single Cerulean donor, Koushik et al. (2009) observed that the FRET transfer rate in this construct exceeded the sum of the rates with the individual acceptors. This effect was named ‘FRET surplus’.

The cause of the enigmatic surplus FRET is not obvious and was discussed in terms of unknown FRET pathways. It can be explained from the statistical nature of the distribution of *κ* ^2^ values of the two competing FRET reactions (Fig. 4b). Assuming a random static or fully dynamic regime, and identical and equidistant acceptors, the sum of both rates would represent an equal chance for each acceptor to engage in FRET upon every donor excitation event. The overall average *κ* ^2^ for each FRET reaction *D* → *A*_1_ and *D* → *A*_2_ is 2/3. However, the *κ* ^2^ values for both D–A pairs will very likely not be the same in time at every donor excitation cycle and the pair with higher *κ* ^2^ value will have a higher chance of coupling. Due to this selection bias, the overall average *κ* ^2^ of a complex will be higher than the value of 2/3 for the D:A = 1:1 condition, increasing the combined FRET rate in the multi-acceptor complex. It is to be expected that this biased ‘sampling’ influence is greatest for the increase from 1 to 2 acceptors and then diminishes. Furthermore, the effect is expected to be largest for equal FRET rates of the individual couples.

**Fig. 5:**
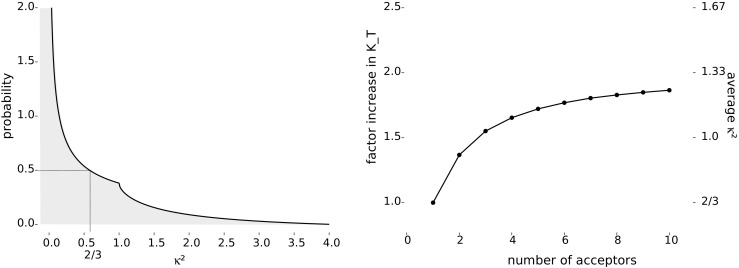
Simulation results illustrating the effect of a selection bias of a higher orientation factor *κ* ^2^ for multi-acceptor FRET. A statistically preferred selection of higher *κ* ^2^ values in case of multi-acceptor conditions increases FRET. *Left panel* Probability distribution of *κ* ^2^ values of a single donor–acceptor pair in dynamic or static random regimes based on the analytical solutions from van der Meer (2002), exhibiting a statistical average of 2/3 (indicated). *Right panel* Simulation of the FRET surplus effect in which the transfer rate for (identical and equidistant) multi-acceptor conditions exceeds the sum of pairs. The probability distribution (*left graph*) was used to create a list of 10,000 entries for *κ* ^2^ values with 0.01 increments. From this, 100,000 items were randomly drawn for each acceptor, of which their averages equal 2/3. FRET events were modeled by drawing the *κ* ^2^ value from the acceptors with a choice likelihood proportional to the magnitude of the *κ* ^2^ values. This produces a list of average *κ* ^2^ values for the different stoichiometries of D:A = 1:1 to 1:10. The average *κ* ^2^ for a donor coupling with multiple acceptors is higher than the canonical 2/3 at the D:A = 1:1 condition

To illustrate the effect of a selection bias of the orientation factor on FRET, we performed a simple simulation (Fig. 5). It is based on the assumption that the probability of selecting a *κ* ^2^ value from multiple identical and equidistant acceptors is proportional to their magnitudes at each excitation cycle (see legend of Fig. 5). This implies a selection bias towards the acceptor with the higher *κ* ^2^ value and, with this, an increase of k_*T*_ exceeding the sum of the individual transfer rates. By increasing the numbers of acceptors, the model shows a first initial steep increase in the average *κ* ^2^, which approaches ∼1.35, corresponding to a doubling of the cumulative FRET rate (≈ 2(*k*_*T*1_ + *k*_*T*2_)). A donor with two equally FRET-competent acceptors already exhibits a ∼40% increase in FRET. This is in excellent agreement with the published data for a D:A = 1:2 construct (Koushik et al. 2009).

## Detection and quantitative analysis of multiplexing FRET

The strength of FRET coupling is usually quantified as a FRET efficiency. The FRET efficiency, typically expressed as a percentage, is a useful and intuitive measure, as it corresponds to the fractional reduction in donor fluorescence intensity, in donor lifetime, and in the number of molecules that undergo FRET. The different FRET microscopy techniques of acceptor photobleaching, fluorescence lifetime imaging microscopy (FLIM), and sensitized emission, the latter optionally combined with spectral unmixing, provide this information and have all been used for the detection of FRET efficiencies in multiplexing experiments.

Nevertheless, in cases where multiple acceptors modify FRET coupling, it might be advantageous to work with the transfer rates, as these are additive. The measured FRET efficiencies as defined in Eq. 6 are not linear and, moreover, even ‘level off’ at higher values due to the inclusion of the transfer rate in the denominator. Consequently, efficiencies are less sensitive than energy rates over a large range of FRET coupling strengths and do not add for the purpose of FRET quantification of the multiplex condition. In the paper that first described the FRET surplus effect (Koushik et al. 2009), FRET efficiencies were transformed to transfer rates to show that the multi-acceptor constructs exhibited a transfer rate that exceeded the sum of the control duplex constructs.

A particularly useful parameter for FRET quantification that we would like to introduce is the ‘normalized transfer rate’ *k* ′ _*T*_ obtained by normalization of the transfer rate to the sum of the radiative and non-radiative rates:

7 $$ k{\prime}_T=\frac{k_T}{\varGamma +{k}_{nr}}={\left(\frac{R_0}{r}\right)}^6 $$

In contrast to transfer rates, the normalized transfer rate can be easily experimentally determined, similar to the FRET efficiency, by acceptor photobleaching microscopy and by FLIM:

8 $$ \begin{array}{l} E=\frac{\varDelta {F}_D}{F_D}=\frac{\varDelta {\tau}_D}{\tau_D}\hfill \\ {} k{\prime}_T=\frac{\varDelta {F}_D}{F_{D A}}=\frac{\varDelta {\tau}_D}{\tau_{D A}}\hfill \end{array} $$

in which Δ*F*_*D*_ denotes donor dequenching upon acceptor photobleaching, *F*_*D*_ is the donor fluorescence after photobleaching, and *F*_*DA*_ is the quenched donor fluorescence in the presence of the acceptor, i.e., before acceptor photobleaching. Similarly, Δ*τ*_*D*_ is the difference between the donor lifetime without (*τ*_*D*_) and with FRET to an acceptor (*τ*_*DA*_).

Most importantly, the normalized transfer rate has the same additive and linear properties as the transfer rate for FRET with multiple acceptors. Furthermore, it exhibits a direct relation between distance and the *R*_0_ distance (Eq. 7), in which a value of 1 denotes a separation distance equal to *R*_0_. A related normalized transfer rate *k*_*T*_/*Γ* has been proposed as a measure for FRET previously (Jares-Erijman and Jovin 2003; Roberti et al. 2011). They adopted this metric for the latter reason. However, we wish to maintain the distance relationship to the classical Förster distances, whereas *k*_*T*_/*Γ* requires the definition of a modified *R*_0_ .

If required, the FRET efficiencies and the normalized transfer rates can be interconverted, for example to calculate the cumulative FRET efficiencies in a multiplexing experiment. The relationship between the FRET efficiency and the normalized transfer rate is given by:

9 $$ \begin{array}{l} E=\frac{k{\prime}_T}{k{\prime}_T+1}\hfill \\ {} k{\prime}_T=\frac{E}{1- E}\hfill \end{array} $$

**Fig. 6:**
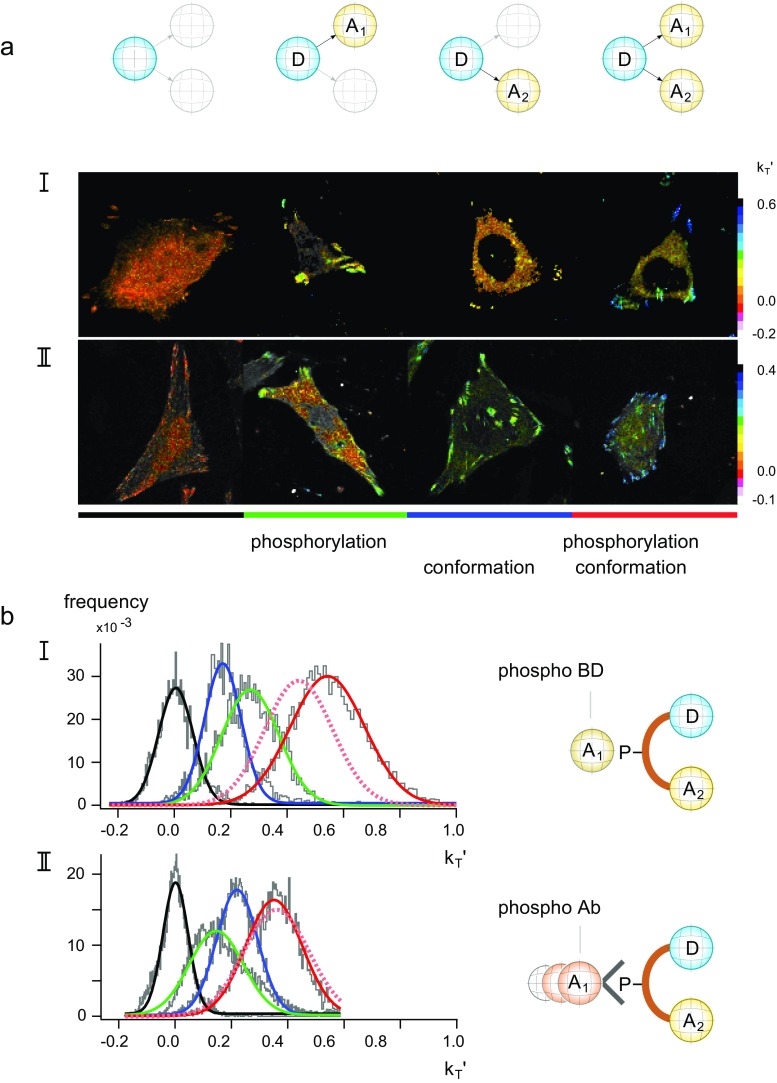
Three-fluorophore FRET experiment for the simultaneous detection of two signaling events: a protein conformation and phosphorylation. **a** Multiplexed FRET was detected between a donor molecule and two shared spectrally identical acceptors. In the FRET assay), the donor D couples to an acceptor A2 in an intramolecular conformational FRET probe (‘FERM sensor’, Papusheva et al. 2009) of the tyrosine kinase FAK, detecting its active conformation, and to a spectrally identical acceptor A1 coupled to a phospho-specific binding protein that recognizes its phosphorylation site Tyr397 (scheme I). **a-I** Representative cells showing FRET for the individual events of conformational activation and phosphorylation of FAK and for their co-occurrence as detected by fluorescence lifetime microscopy. **b-I** Cumulative normalized *k*_*T*_ distributions and their Gaussian fits of pixel values in all focal adhesion sites of several cells (*n* = 5). Focal adhesions are the primary sites of FAK activation (*green curve* phosphorylated FAK; *blue curve* conformationally active FAK; *red curve* conformationally active and phosphorylated FAK; *black curve* FAK control). Normalized *k*_*T*_ distributions were determined according to Eq. 8. The experimental sum of *k*_*T*_ value distributions of the individual events (*red solid curve*) exceeds the predicted sum (*red dashed curve*) by 20% due to the ‘surplus’ effect. Scheme II: The simultaneous detection of a FAK conformation and phosphorylation using anti-phospho-specific Tyr397 antibody staining instead of a fluorescent protein-based genetic approach, as used in scheme I. **a-II** Fluorescence lifetime detection of representative cells for the individual events and their co-occurrence. **b-II** The cumulative normalized *k*_*T*_ distributions and their Gaussian fits for several cells (*n* = 5), with color-coding according to **b-I**. Here, the experimental sum of the curves matches the predicted distribution, indicating the presence of the ‘antenna’ but not the ‘surplus’ effect. This type of experiment serves as a control for the ‘surplus’ effect by virtue of the acceptor-to-donor excess in the multiple-acceptor–labeled antibody complex, in which the surplus effect already acts. An increase due to the addition of one acceptor becomes negligible

Especially, the surplus effect presents a difficulty for FRET quantification. Together with the antenna effect, it, however, presents an elegant diagnostic tool for the unequivocal determination of complex (or oligomer) formation. When a donor-labeled component interacts with two different acceptor-labeled components A_1_ and A_2_, and the biological question relates to the DA_1_A_2_ complex formation with possible DA_1_ and DA_2_ intermediates, the antenna and surplus effects exclusively operate on the ternary complex DA_1_A_2_. When FRET exceeds the sum of DA_1_ and DA_2_ measurements, ternary complex formation is a fact. Both the antenna and surplus effects can be clearly detected in the donor channel as donor yield and lifetime changes and can, therefore, be effectively read out by donor-only methods as acceptor photobleaching and FLIM (Fig. 6).

In contrast, in multiplexing experiments based on sensitized emission measurements, the energy transfer is detected from changes in acceptor fluorescence. Usually, binary interactions between the individual donor acceptor pathways (DA_1_ and DA_2_) are sampled in such experiments. However, the emission detected for each of the acceptor channels is effectively reduced, as the energy transferred from the shared donor to the additional acceptor does not contribute to the measured sensitized emission (Eq. 10). In analogy to Eq. 6, the FRET efficiencies in sensitized emission are:

10 $$ {E}_{se, A1}^{A2}=\frac{k_{T1}}{k_{T1}+{k}_{T2}+\Gamma +{k}_n}<{E}_{se, A1}=\frac{k_{T1}}{k_{T1}+\Gamma +{k}_n} $$

Consequently, the FRET efficiency measured by sensitized emission of acceptor 1 (*E*_*se, A1*_) is lowered due to the ‘antenna’ effect by the presence of acceptor 2, indicated by the suffix A2 (*E*_*se,A1*_^*A2*^). The same holds true for sensitized emission measured from acceptor 2. For the same reason, the surplus effect will result in a further reduction of the FRET efficiency, leading to its underestimation.

The FRET quantification of sensitized emission measurements using different acceptors has been solved and used for the study of multi-protein interactions. We refer to several excellent papers on this topic: Fried et al. (2014), Pauker et al. (2012), Scott and Hoppe (2016), Sun et al. (2010), and Wallrabe et al. (2015). Sensitized emission FRET with multiple identical acceptors, e.g., in oligomerization experiments, on the other hand, behaves similar to donor FRET measurements.

In conclusion, both the donor-only and the sensitized emission approaches provide relevant information and both have underlying antenna and surplus effects. However, in sensitized emission FRET, the surplus effect is not experimentally accessible, as a measurement of sensitized emission on the combined non-identical acceptors is typically not performed. This situation is different in the donor FRET methods, where the transfer of energy to all acceptors combined is measured.

## Conclusion

Sophisticated Förster resonance energy transfer (FRET) multiplexing schemes are increasingly being used to elucidate multiple protein interactions in single cells. These assays hold the promise to solve the dynamic connectome of strategic sets of proteins, for instance to understand decision points in signaling events, as well as the stoichiometry of multimeric protein complexes. However, their quantitation is complicated by the fact that FRET between a single donor and multiple acceptors behaves differently from the known one-on-one condition. In addition to a predictable ‘antenna’ effect that is based on the summation of the transfer rates that act on the donor, quantification is confounded by a ‘surplus’ effect, likely caused by the statistical selection of a more favorable relative dipole orientation among the acceptors. These effects have an influence on the quantification of FRET independent of the microscopy method used. At the same time, they can be taken as powerful diagnostic indicators for true FRET multiplexing of donor molecules. As acceptor-based FRET measurements predominantly provide information on the coupling strength to a specific spectrally distinct acceptor, and donor FRET measurements, especially FLIM, provide easy access to rates that permit the evaluation of the surplus effect, we expect to see an increase in multimodal, spectrally resolved, and quantitative FRET microscopy approaches in the future that incorporate the strengths of the individual methods.

## References

[CR1] Aye-Han N-N, Allen MD, Ni Q, Zhang J (2012). Parallel tracking of cAMP and PKA signaling dynamics in living cells with FRET-based fluorescent biosensors. Mol Biosyst.

[CR2] Bastiaens P, de Beus A, Lacker M, Somerharju P, Vauhkonen M, Eisinger J (1990). Resonance energy transfer from a cylindrical distribution of donors to a plane of acceptors. Location of apo-B100 protein on the human low-density lipoprotein particle. Biophys J.

[CR3] Dale RE, Eisinger J, Blumberg WE (1979). The orientational freedom of molecular probes. The orientation factor in intramolecular energy transfer. Biophys J.

[CR4] Ding Y, Ai HW, Hoi H, Campbell RE (2011). Förster resonance energy transfer-based biosensors for multiparameter ratiometric imaging of Ca2+ dynamics and caspase-3 activity in single cells. Anal Chem.

[CR5] Fábián ÁI, Rente T, Szöllősi J, Mátyus L, Jenei A (2010). Strength in numbers: effects of acceptor abundance on FRET efficiency. ChemPhysChem.

[CR6] Förster T (1946). Energiewanderung und Fluoreszenz. Naturwissenschaften.

[CR7] Förster T (1948). Zwischenmolekulare Energiewanderung und Fluoreszenz. Ann Phys.

[CR8] Förster T (1951). Fluoreszenz organischer Verbindungen.

[CR9] Fried S, Reicher B, Pauker MH, Eliyahu S, Matalon O, Noy E, Chill J, Barda-Saad M (2014). Triple-color FRET analysis reveals conformational changes in the WIP-WASp actin-regulating complex. Sci Signal.

[CR10] Galperin E, Verkhusha VV, Sorkin A (2004). Three-chromophore FRET microscopy to analyze multiprotein interactions in living cells. Nat Methods.

[CR11] Grant DM, Zhang W, McGhee EJ, Bunney TD, Talbot CB, Kumar S, Munro I, Dunsby C, Neil MAA, Katan M, French PMW (2008). Multiplexed FRET to image multiple signaling events in live cells. Biophys J.

[CR12] Jares-Erijman EA, Jovin TM (2003). FRET imaging. Nat Biotechnol.

[CR13] Koushik SV, Blank PS, Vogel SS (2009). Anomalous surplus energy transfer observed with multiple FRET acceptors. PLoS One.

[CR14] Kuhn H, Weissberger A, Rossiter BW (1972). Spectroscopy of monolayer assemblies, part 1, principles and applications. Physical methods of chemistry.

[CR15] Laviv T, Kim BB, Chu J, Lam AJ, Lin MZ, Yasuda R (2016). Simultaneous dual-color fluorescence lifetime imaging with novel red-shifted fluorescent proteins. Nat Methods.

[CR16] Maliwal BP, Raut S, Fudala R, D’Auria S, Marzullo VM, Luini A, Gryczynski I, Gryczynski Z (2012). Extending Förster resonance energy transfer measurements beyond 100 Å using common organic fluorophores: enhanced transfer in the presence of multiple acceptors. J Biomed Opt.

[CR17] Niino Y, Hotta K, Oka K (2009). Simultaneous live cell imaging using dual FRET sensors with a single excitation light. PLoS One.

[CR18] Niino Y, Hotta K, Oka K (2010). Blue fluorescent cGMP sensor for multiparameter fluorescence imaging. PLoS One.

[CR19] Ouyang M, Huang H, Shaner NC, Remacle AG, Shiryaev SA, Strongin AY, Tsien RY, Wang Y (2010). Simultaneous visualization of protumorigenic Src and MT1-MMP activities with fluorescence resonance energy transfer. Cancer Res.

[CR20] Papusheva E, Mello de Queiroz F, Dalous J, Han Y, Esposito A, Jares-Erijman EA, Jovin TM, Bunt G (2009). Dynamic conformational changes in the FERM domain of FAK are involved in focal-adhesion behavior during cell spreading and motility. J Cell Sci.

[CR21] Pauker MH, Hassan N, Noy E, Reicher B, Barda-Saad M (2012). Studying the dynamics of SLP-76, Nck, and Vav1 multimolecular complex formation in live human cells with triple-color FRET. Sci Signal.

[CR22] Piljic A, Schultz C (2008). Simultaneous recording of multiple cellular events by FRET. ACS Chem Biol.

[CR23] Raicu V (2007). Efficiency of resonance energy transfer in homo-oligomeric complexes of proteins. J Biol Phys.

[CR24] Roberti MJ, Giordano L, Jovin TM, Jares-Erijman EA (2011). FRET imaging by k(t)/k(f). ChemPhysChem.

[CR25] Scott BL, Hoppe AD (2016). Three-dimensional reconstruction of three-way FRET microscopy improves imaging of multiple protein–protein interactions. PLoS One.

[CR26] Sergeeva TF, Shirmanova MV, Zlobovskaya OA, Gavrina AI, Dudenkova VV, Lukina MM, Lukyanov KA, Zagaynova EV (2017). Relationship between intracellular pH, metabolic co-factors and caspase-3 activation in cancer cells during apoptosis. Biochim Biophys Acta.

[CR27] Shimozono S, Hosoi H, Mizuno H, Fukano T, Tahara T, Miyawaki A (2006). Concatenation of cyan and yellow fluorescent proteins for efficient resonance energy transfer. Biochemistry.

[CR28] Singh DR, Mohammad MM, Patowary S, Stoneman MR, Oliver JA, Movileanu L, Raicu V (2013). Determination of the quaternary structure of a bacterial ATP-binding cassette (ABC) transporter in living cells. Integr Biol.

[CR29] Stoneman MR, Paprocki JD, Biener G, Yokoi K, Shevade A, Kuchin S, Raicu V (2016). Quaternary structure of the yeast pheromone receptor Ste2 in living cells. Biochim Biophys Acta.

[CR30] Sun Y, Wallrabe H, Booker CF, Day RN, Periasamy A (2010). Three-color spectral FRET microscopy localizes three interacting proteins in living cells. Biophys J.

[CR31] Uphoff S, Holden SJ, Le Reste L, Periz J, van de Linde S, Heilemann M, Kapanidis AN (2010). Monitoring multiple distances within a single molecule using switchable FRET. Nat Methods.

[CR32] van der Meer BW (2002). Kappa-squared: from nuisance to new sense. Rev Mol Biotechnol.

[CR33] van der Meer BW, Medintz I, Hildebrandt N (2013). Förster theory. FRET—Förster resonance energy transfer: from theory to applications.

[CR34] Walczewska-Szewc K, Bojarski P, d’Auria S (2013). Extending the range of FRET—the Monte Carlo study of the antenna effect. J Mol Model.

[CR35] Wallrabe H, Sun Y, Fang X, Periasamy A, Bloom GS (2015). Three-color confocal Förster (or fluorescence) resonance energy transfer microscopy: quantitative analysis of protein interactions in the nucleation of actin filaments in live cells. Cytom Part A.

[CR36] Warren SC, Margineanu A, Katan M, Dunsby C, French PMW (2015). Homo-FRET based biosensors and their application to multiplexed imaging of signalling events in live cells. Int J Mol Sci.

[CR37] Watrob HM, Pan C-P, Barkley MD (2003). Two-step FRET as a structural tool. J Am Chem Soc.

[CR38] Woehler A (2013). Simultaneous quantitative live cell imaging of multiple FRET-based biosensors. PLoS One.

[CR39] Wouters FS, Kubitscheck U (2013). Förster resonance energy transfer and fluorescence lifetime imaging. Fluorescence microscopy: from principles to biological applications.

